# Cost-informed Bayesian reaction optimization†

**DOI:** 10.1039/d4dd00225c

**Published:** 2024-10-01

**Authors:** Alexandre A. Schoepfer, Jan Weinreich, Ruben Laplaza, Jerome Waser, Clemence Corminboeuf

**Affiliations:** a Laboratory for Computational Molecular Design, Institute of Chemical Sciences and Engineering, École Polytechnique Fédérale de Lausanne (EPFL) 1015 Lausanne Switzerland clemence.corminboeuf@epfl.ch; b Laboratory of Catalysis and Organic Synthesis, Institute of Chemical Sciences and Engineering, École Polytechnique Fédérale de Lausanne (EPFL) 1015 Lausanne Switzerland jerome.waser@epfl.ch; c National Center for Competence in Research-Catalysis (NCCR-Catalysis), École Polytechnique Fédérale de Lausanne 1015 Lausanne Switzerland

## Abstract

Bayesian optimization (BO) is an efficient method for solving complex optimization problems, including those in chemical research, where it is gaining significant popularity. Although effective in guiding experimental design, BO does not account for experimentation costs: testing readily available reagents under different conditions could be more cost and time-effective than synthesizing or buying additional ones. To address this issue, we present cost-informed BO (CIBO), an approach tailored for the rational planning of chemical experimentation that prioritizes the most cost-effective experiments. Reagents are used only when their anticipated improvement in reaction performance sufficiently outweighs their costs. Our algorithm tracks available reagents, including those recently acquired, and dynamically updates their cost during the optimization. Using literature data of Pd-catalyzed reactions, we show that CIBO reduces the cost of reaction optimization by up to 90% compared to standard BO. Our approach is compatible with any type of cost, *e.g.*, of buying equipment or compounds, waiting time, as well as environmental or security concerns. We believe CIBO extends the possibilities of BO in chemistry and envision applications for both traditional and self-driving laboratories for experiment planning.

## Abstract 2

Cost-informed Bayesian optimization (CIBO) is a method for chemical reaction optimization that incorporates reagent and experimentation costs, to significantly reduce expenses while maintaining efficiency.

## Graphical abstract

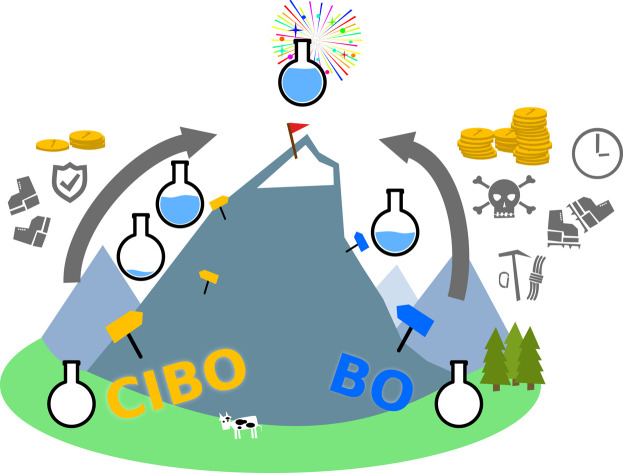

## 1 Introduction

Reaction optimization is a challenging task that is often tackled “one factor at a time” by sequentially optimizing individual parameters such as catalyst, temperature, or additives. While this strategy simplifies the problem, it remains both time and resource-intensive. Moreover, promising combinations of parameters may be overlooked. For instance, an additive and ligand, which were discarded when tested individually, may yield optimal results when combined.

As an alternative, data-driven computational tools, such as machine learning (ML), have recently been used to guide experimental effort aimed at achieving the best possible performance by predicting reaction yield or selectivity from substrates, catalysts, and reaction conditions.^1–6^ Amongst different ML frameworks, Bayesian optimization (BO) is ideally suited for this task.^7,8^ Given initial data, BO leverages predictions and their corresponding uncertainties to suggest the next experiments evaluated as the most promising. As such, BO-driven reaction optimization has seen notable success in the last few years, especially in automated laboratory and high-throughput experimentation (HTE) settings.^3,9–16^ In such situations, all materials being considered (*i.e.*, substrates, catalysts, additives, solvents) are typically procured prior to experimentation, at which point BO is used to identify the best reagents and reaction conditions.^1,3,17,18^

Yet, the implementation of BO and other ML frameworks in more traditional (*i.e.*, not high-throughput) laboratories is still limited.^19–22^ In these settings, defining and acquiring all necessary materials prior to the optimization, especially when dealing with unexplored chemistry, is not ideal. A further complication is that classical BO methods usually attribute the same cost to all suggested experiments. In reaction optimization, where *e.g.*, catalyst ligands and reaction conditions have to be adjusted simultaneously, this assumption is also unsuitable as the cost of an experiment (in terms of money, time investment, or risk) can vary drastically depending on whether a ligand is already available in the laboratory, commercially available, reported in the literature, or has never been synthesized before. Thus, experiments suggested by BO may be impractical or even non-feasible and human filtering might be necessary. On the other hand, testing already available ligands with different reaction conditions may yield a comparable improvement with lower cost, especially in the preliminary stages of reaction optimization when less is known about the chemistry.

**Fig. 1:**
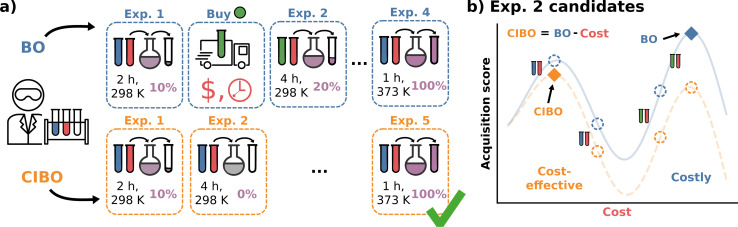
Overview of standard BO (blue) *vs.* cost-informed Bayesian optimization (CIBO, orange) for yield optimization. (a) BO recommends purchasing more materials. Meanwhile, CIBO balances purchases with their expected improvement of the experiment, at the cost of performing more experiments (here five *vs.* four). (b) A closer look at the two acquisition functions of BO and CIBO for the selection of experiment two. In CIBO, the BO acquisition function is modified to account for the cost by subtracting the latter. Following the blue BO curve, the next experiment to perform uses green and red reactants (corresponding to the costly maximum on the right). Subtracting the price of the experiments results in the orange CIBO curve, which instead suggests the more cost-effective experiment on the left (blue and red reactants).

To overcome this limitation, we introduce cost-informed Bayesian optimization (CIBO), a BO framework that incorporates cost into the decision-making process to achieve practical and rational batch experimentation planning. CIBO optimizes a single objective by incorporating contextual information into the decision-making process. For instance, if two equally informative experiments are possible, CIBO suggests the one with the lower cost (Fig. 1). Additionally, it maintains a digital inventory that dynamically updates experiment costs based on the resources currently available. For example, once a ligand is purchased, it can be used for several experiments.

Previous methods aimed at optimizing reaction cost and performance relied on multi-objective approaches or included constraints on the overall cost.^16,18,23,24^ Multi-objective methods typically balance competing objectives like yield, selectivity, and cost, often using complex scalarizing methods to manage trade-offs (*e.g.*, Pareto fronts). CIBO also contrasts with (non-multi-objective) methods that have been proposed in chemistry (*vide infra*), as well as in other fields, such as cost-aware BO in computer science.^25–27^ Those are based on different strategies and include, for instance, contextual BO,^28^ addressing environmental effects, as well as direct modifications of the acquisition function.^29–31^ Methods that favor low-cost experiments for a given budget have been suggested,^32–36^ but with the costs kept fixed throughout the optimization. Resource management optimization is a related problem that has been investigated, albeit not with BO.^37–40^ More recent works have focused on the cost of changing between different experimental setups to account for the associated expenses^41–48^ or on human-in-the-loop strategies.^49^ Finding cost-efficient routes in the context of retro-synthesis planning has also been explored^50–54^ although the focus is placed on proposing plausible and practical pathways rather than on optimizing the conditions of individual reactions.

To our knowledge, a reaction optimization framework accounting for both the cost of experiments contextually and the fact that these costs are dynamically updated over time has not been introduced. Here, we demonstrate the performance of CIBO using two HTE datasets of Pd-catalyzed reactions and find that, despite occasionally requiring additional experiments to match standard BO, the overall cost of the optimization is significantly reduced. Our benchmarks evaluate cost using the price of commercially available reagents. However, CIBO is compatible with any cost definition, such as the number of steps or estimated time required for synthesizing reported ligands, as well as sustainability metrics for solvents or compounds. In the latter case, the platform will prioritize options with lower environmental impact.^55–57^ Overall, CIBO promises an efficient and sustainable alternative to existing design-of-experiment methods.^58,59^

## 2 Methods

### 2.1 Cost-informed Bayesian optimization

BO is an effective method for optimizing noisy functions that are expensive or time-consuming to evaluate.^60^ At its core lies a surrogate model that predicts the value of the function (mean *μ*) and the uncertainty of the prediction (standard deviation *σ*). Acquisition functions are used to identify promising experiments in the design space.^61^ This is done by considering all possible experiments and weighting their potential to maximize *μ*, balancing the trade-off between exploring uncertain areas of the design space (exploration) and exploiting areas known to yield high values (exploitation). In addition to performing one experiment at a time, batch BO proposes a joined set (*i.e.*, a batch of experiments) which provides the largest expected improvement when performed in parallel.

Given the previous definition, BO does not account for the varying costs of the resources involved in the optimization. Instead of buying or synthesizing additional substances, chemists may first choose to vary easily controllable conditions (*e.g.*, temperature, reaction time), resulting in lower costs and a better-informed decision before acquiring additional compounds. Finding the best experimental conditions for the smallest budget is different from identifying the best value–cost trade-off in the optimized reaction. The latter is relevant when large amounts of the compounds involved need to be acquired repeatedly: *e.g.*, large-scale synthesis. The former, and current case, is relevant when the budget (in terms of cost, time, or other) for the experimentation campaign itself is important.

Our method, cost-informed Bayesian optimization (CIBO), balances minimizing experimentation cost with maximizing measured improvement (see Fig. 1). It results in experiments that more closely mimic the optimization process in a chemistry lab, only acquiring a compound if the expected improvement justifies the cost. CIBO stands out from standard BO by promoting the search for more cost-effective experiments while not constraining its search space (*i.e.*, an expensive ligand may still be selected if its expected improvement is justified). We account for experimentation costs by including them in the acquisition function,^62,63^ scaling the cost adjustment with the expected improvement values. Given the set of all possible experiments {*e*}, we use the batch noisy expected improvement (qNEI) acquisition function {*α*_*e*_}, computed from the predicted *μ* and *σ* from the surrogate model to determine the next batch 
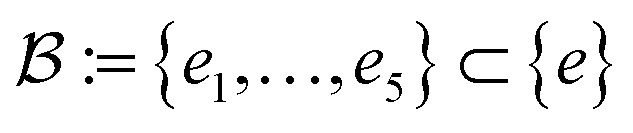
 for each iteration.^64,65^ Here *e*_*j*_ is the *j*-th experiment in batch 
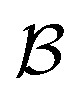
 with acquisition function value *α*_*j*_ ≡ *α*_*e*_j__. Note that there is exactly one *α*_*j*_ per experiment *e*_*j*_. For a batch of experiments, here *N*_*e*_ = 5 per batch, we consider the current cost of each compound involved. As user input, only the compound prices per gram, per mol, per bottle, or other user-defined costs *p*_*j*_ are required.

For simplicity, we cover the case of one compound *j* per experiment *e*_*j*_. Batches are ordered with respect to the norm of 
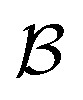
, defined by the sum of the acquisition function values *α*_*i*_ in each batch,1
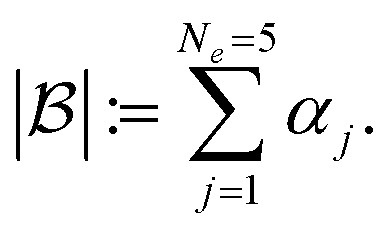
In standard batch BO, the batch with the highest rank (*i.e.*, the highest expected improvement, represented by the blue line in Fig. 1b) is chosen for the next set of experiments, as it offers the best combination of expected improvement and cost. In CIBO, we modify qNEI of each experiment *e*_*j*_ by subtracting a term^47,62,63^ proportional to the cost 
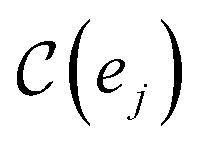
 as follows:2
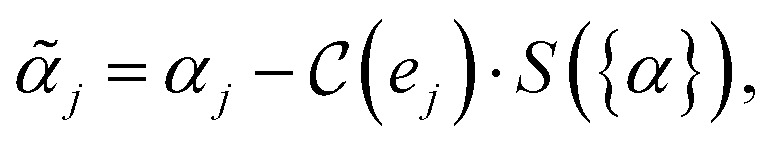
where 
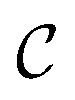
 probes whether compound *j* was bought at price *p*_*j*_ in a previous iteration or added to the same batch before, *i.e.*,3
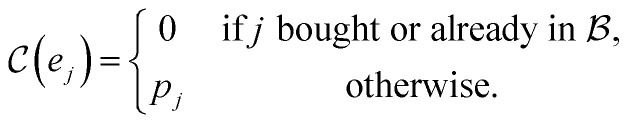
This means that the cost is zero when *j* was obtained in some previous iteration or appeared in the same batch 
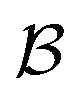
 but under different experimental conditions. For simplicity, throughout this work we assume that compounds in the inventory are never exhausted. However, our framework could be set to deduct exact quantities from the inventory until nothing remains.

In eqn (2), we introduced a scaling function *S* that depends on all acquisition function values {*α*} evaluated on all experiments not yet included in the surrogate model and the current prices {*p*} for each ligand,

4 *S*:= *λ*·max{*α*}/avg{*p*},

to update the magnitude of the prices that enter eqn (2) such that the maximum value of the subtracted term has a comparable value as the largest acquisition function value. The purpose of this rescaling is to balance cost with the exploration–exploitation trade-off defined by the original qNEI acquisition function term *α*. The scaling function is updated after each iteration to ensure adapting to the current costs and acquisition function values. Additionally, the scaling function removes the cost units. Finally, *λ* controls the weighting between yield optimization and reducing the costs of the optimization. For *λ* = 0 CIBO is equivalent to normal BO, for *λ* > 0 costs are taken into account, increasing *λ* puts more weight on reducing the costs. If not mentioned otherwise, we set *λ* = 1.0 (see ESI Section S4 for additional details†).

After computing the modified acquisition values for each potential experiment in a batch *α̃*_1_, … *α̃*_5_, we evaluate the updated norm value,5
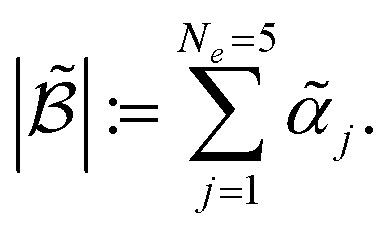
This corresponds to the orange line in Fig. 1b, which differs from the blue line depending on the scaled cost term of eqn (2). The batch with maximal value 
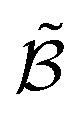
, offering the best cost–benefit ratio, is then selected for the next iteration.

Note that our approach does not penalize exploration *per se*, but rather favors the most inexpensive ways to explore before committing to higher-cost experiments. Further details of our implementation are available in the ESI Section S1.†

### 2.2 Datasets and models

**Fig. 2:**
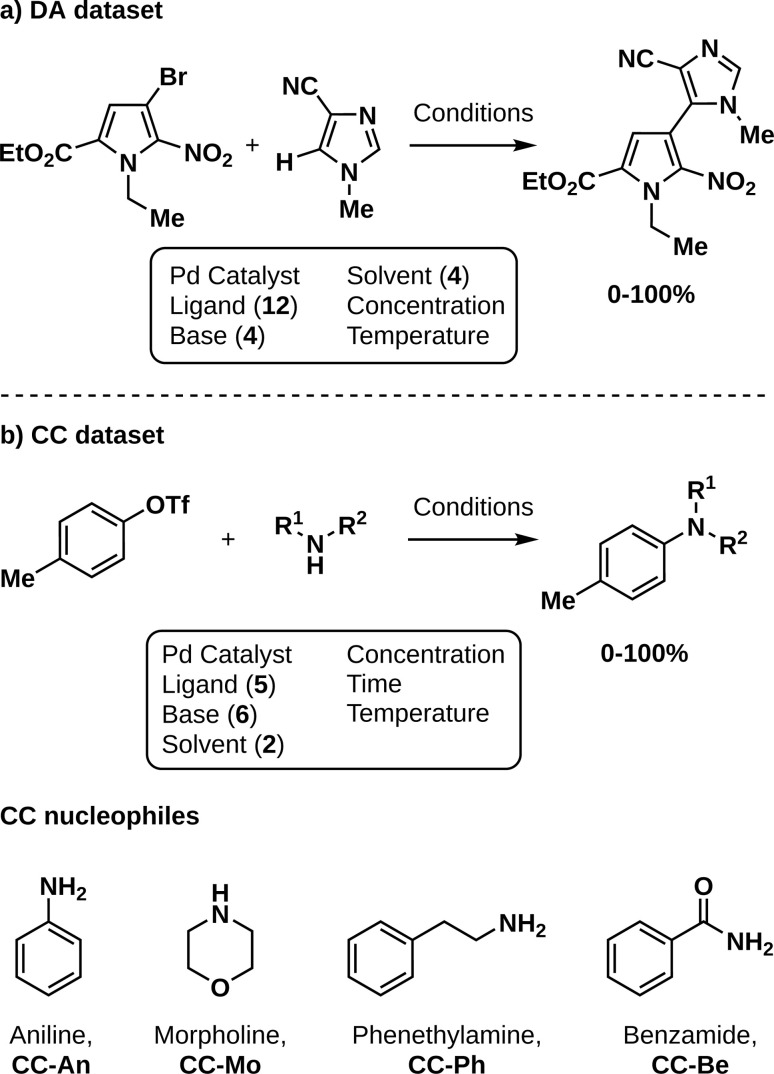
Reaction schemes of the two datasets used in this work. (a) Direct arylation (DA) with yields ranging from 0–100%.^3^ (b) Cross-coupling (CC) with yields ranging from 0–100%.^17^ The four nucleophiles explored in the latter dataset are depicted below, each leading to a subset (An, Mo, Ph, Be).

To demonstrate the potential of CIBO, we attempt to maximize reaction yield in the most cost-efficient manner for two literature datasets: Pd-catalyzed direct C–H arylation (DA, Fig. 2a)^3^ and Pd-catalyzed Buchwald–Hartwig cross-couplings of amine nucleophiles using a droplet platform (CC, Fig. 2b).^17^ Being the most expensive element of the optimization, CIBO should avoid using unnecessary ligands to reach a high yield.

In both cases, we use Gaussian process regression with a Jaccard–Tanimoto kernel (see ESI Section S2 for more details†) as the surrogate model in the optimization. The batch size is always fixed to five,^3,18^ but we note any other value could be used according to the preferred experimental setup. As the cost, we use the price per gram of material (dollars per gram $/g, see ESI Section S3 for details†), since chemical suppliers provide most chemicals gram-wise.

For consistency and to make the task challenging, we start the optimization using models trained only on a small subset of experiments performed with chemicals with low overall cost or performance (iteration zero, *vide infra*). The cost of the initial experiments is included in the total cost. For simplicity, we assume that we never run out of chemicals in the inventory, which applies both to the reagents used in the initial experiments and to newly acquired ones. The results are averaged over 100 separate runs using the same initialization.^66^

The DA dataset consists of 1728 measurements where the monophosphine ligand, base, solvent, concentration, and temperature are varied to optimize the formation of one product. The cost of chemicals was reported previously^18^ and was converted to dollars per gram for this work. We considered concentration and temperature to be variables with no additional cost. In general, the costs of the base and solvent are negligible compared to the ligand. The optimization begins with the surrogate model trained exclusively on the 144 experiments in the dataset that use the dimethylphenylphosphine ligand, which is inexpensive and the worst-performing ligand overall. The minimal spending to obtain the best results corresponds to $144 for this dataset.

For the CC dataset, four different amine nucleophiles, aniline (An), morpholine (Mo), phenethylamine (Ph), and benzamide (Be), are each coupled with *p*-tolyl triflate yielding different products over 363 reactions (around 90 per nucleophile). Each is considered an individual reaction in which the ligand (precatalyst), base (concentration and equivalents), solvent, temperature, and time all change.^23^ The prices for all chemicals involved were obtained from supplier websites and converted to dollars per gram if necessary. Concentrations, equivalents, temperature, and time are varied without additional cost. On average, nine experiments are used at the start of each optimization. For the Ph and Be subsets of CC we initialize using tBuXPhos as a ligand (for the precatalyst) and DBU as a base, the cheapest combination. For An, we chose to initialize with EPhos and TEA as the cheapest initialization would have resulted in perfect yield. For Mo no measurements with tBuXPhos were performed. Thus, we initialize with tBuBrettPhos and DBU. For the CC experiments, the cost of the solvents and bases were also taken into account, since the dataset contains only a few different ligands per nucleophile. The minimal spending to obtain the best results is $69 for An, $1382 for Mo, $97 for Ph, and $591 for Be respectively. A full list of chemical abbreviations is presented in Table S1.†

## 3 Results

### 3.1 Direct arylation

**Fig. 3:**
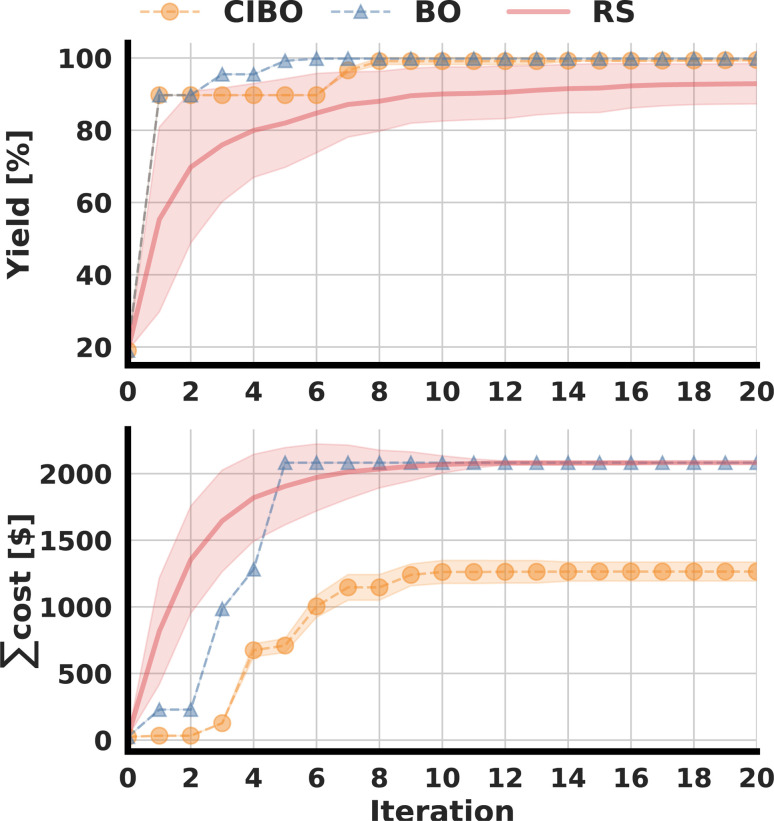
The figure compares cost-informed Bayesian optimization (CIBO, orange), standard BO (blue), and random sampling (RS, red) for the DA dataset over 100 averaged runs. Shaded areas indicating the standard deviation, representing the variability across runs. The best-obtained yield in each batch iteration is shown in the top panel, and the sum spent to acquire the ligands is shown below.

Following the original publication, the optimization was run for 20 total iterations (100 experiments).^3^ The best-observed yield for several batched iterations, as well as the total amount spent, are shown in Fig. 3. CIBO achieves a yield of over 99% after eight iterations, compared to five iterations in standard BO. However, the total cost of running the five iterations (25 experiments) suggested by BO is, on average $1592, whereas the cost of running the eight iterations proposed by CIBO (40 experiments) is, on average, $1156, 38% less. After 20 iterations, standard BO bought all possible ligands (12), amounting to a cost of $2082, while CIBO bought two fewer ligands (10), for a total spending of $1221, thereby saving more than 40%. Considering the error of the surrogate model (see ESI Section S1†) it is prudent not to buy additional ligands after reaching a yield above 99%. Following this logic, past that point (in the tenth iteration), CIBO suggests optimizing reaction conditions instead of buying expensive ligands.

**Fig. 4:**
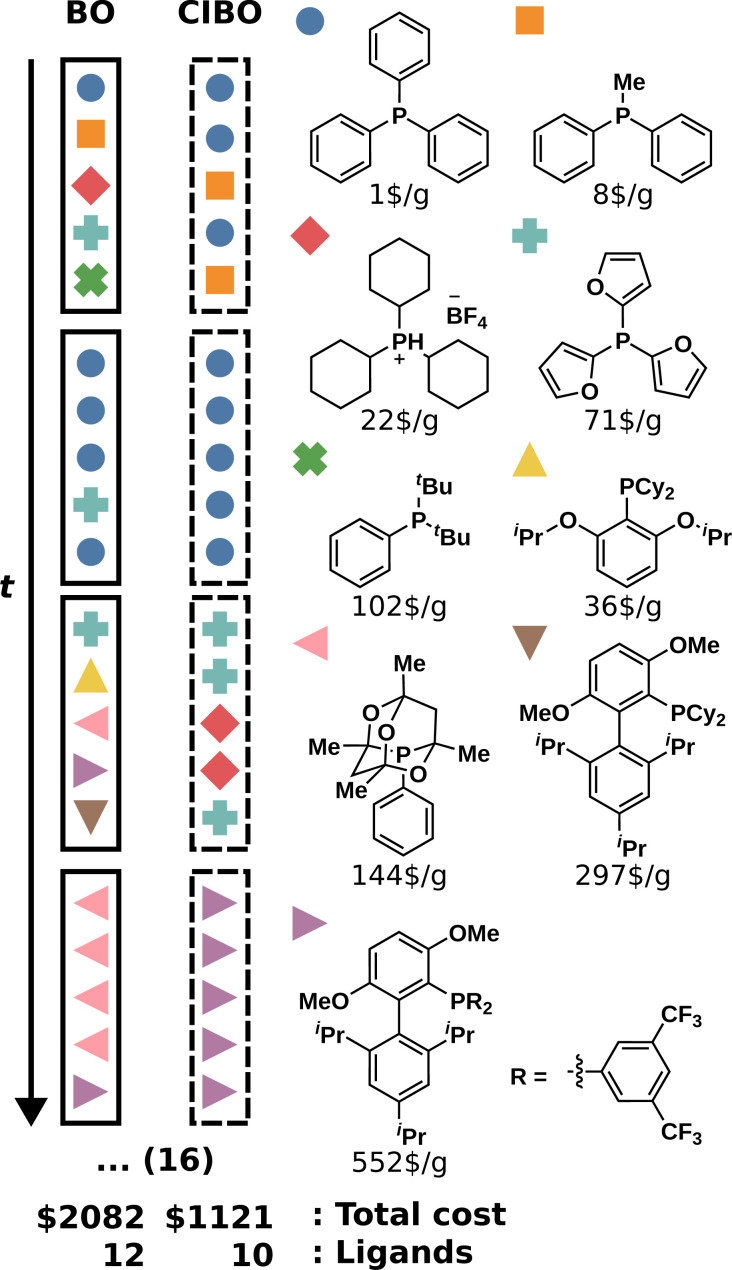
Composition of the four first batches of ligands with different reaction conditions for the DA dataset. We compare the total cost and number of ligands after 20 (16 more) iterations for BO and CIBO. Every colored symbol corresponds to one ligand shown on the right respectively.

To study the difference in experimentation planning between BO and CIBO, we visualize the ligand batch composition of the first four iterations (see Fig. 4) for one of the five repetitions. CIBO batches are less diverse than BO in terms of ligands: CIBO suggests at most two different ligands per batch. In the first iteration, BO suggests acquiring five ligands in addition to dimethylphenylphosphine, compared to only two for CIBO. For a detailed analysis of the effect of initialization, see ESI Section S5.†

Finally, we investigated the influence of the weighting factor *λ* (see eqn (4)) on the results and found the expected behavior: increasing *λ* substantially decreases the costs and *vice versa*. However, this comes with a trade-off in yield optimization and an additional increase in the number of iterations required to obtain the yield for a smaller cost weighting (see ESI Section S4 for details†).

### 3.2 Cross-coupling

The top row shows the best yield found as a function of the batch iteration, and the bottom row displays the cumulative costs. The black dotted line in the top row indicates the target yield at 70%. The resulting terminal iteration (vertical black line) in the bottom row indicates the total budget spent with CIBO.

The dataset is split into subsets of the four different amine nucleophiles (see Fig. 2b) resulting in four distinct optimization problems. As before, we consider the best-observed yield and total cost per iteration. The total dollar amount spent, listed in the first row of Table 1, depends on the nucleophile subset, since not all ligand (precatalyst)/base combinations were tested experimentally for each nucleophile. Due to the limited available data, the optimization is continued until the experimental data is exhausted, and the results are averaged over 100 different runs.

**Table 1:** Comparison of the amounts spent with CIBO and classical BO for each nucleophile subset of CC experiments. The first row lists the total cost of all compounds that could be acquired, which equals the amount spent by classical BO. The subsequent rows show the amount spent with CIBO and the amount and percentage saved compared to classical BO to achieve a yield above 70%. The last row indicates how many compounds did not have to be obtained out of the available ones

	An	Mo	Ph	Be
Cost all reagents	$2474	$2105	$2170	$2144
CIBO spent	$1124	$2105	$142	$690
Saved wrt. BO	$1350 (**54%**)	0	$2028 (**93%**)	$1454 (**68%**)
Saved reagents	5/9	0/7	4/8	3/8

**Fig. 5:**
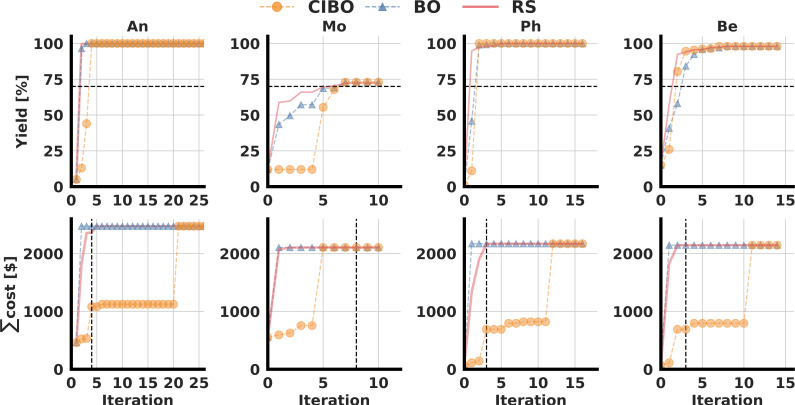
Yield optimization for the CC dataset with four different nucleophiles (An, Mo, Ph, Be). We compare cost-informed Bayesian optimization (CIBO, orange), Bayesian optimization (BO, blue), and random sampling (RS, red). Average curves over 100 runs are shown. Error bars are not shown due to the small variability in the selection of experiments across runs.

As shown in the bottom row of Fig. 5 standard BO suggests buying all available ligand and base combinations immediately following the first iteration – irrespective of their costs. CIBO acquires less expensive reagents first and recommends experiments under varying conditions before finally buying all reagents – if no other experiments are left in the dataset. By inspecting the amount of money spent *versus* the number of iterations, Fig. 5, it is apparent that CIBO suggests more expensive molecules only as a last option. If possible, CIBO optimizes the yield based on reagent/condition combinations that can be performed with minimal or zero additional cost. The case of Be is illustrative: after iteration four, over 30 experiments are performed without acquiring any additional reagents, resulting in a cost plateau. Similar observations are made for An and Ph (Fig. 5a and c) where the optimization achieved a yield of 100% after only a few iterations, followed by iterations where CIBO does not acquire additional reagents.

The goal of every experimentation series always depends on the context. We define a stopping criterion when at least 70% yield is achieved, as this was deemed to be high yield in the original CC publication.^17^ In Table 1 we show the cost and reagent savings compared to standard BO when using this criterion. In all cases, except for Mo, the cost is reduced by over 50%, and fewer compounds were acquired.

For Mo, CIBO performs as well as BO in terms of yield optimization for the same cost, but only because the highest performing ligand is also the most expensive that must be acquired to achieve high yield. For Ph, CIBO requires no new ligands but manages to find a perfect yield by buying a much cheaper base (BTTP). This results in a savings of 93% compared to the standard BO experiments that acquire all ligands in a single step.

## 4 Conclusion

We introduced cost-informed Bayesian optimization (CIBO), a variant of BO that balances cost and ease of experimentation with a global optimization objective. Akin to BO, the algorithm retains the flexibility to identify the most promising experiments but takes a more cost-efficient optimization path by updating the acquisition function according to the current inventory status.

This work focuses on limiting the economic cost engendered by the purchase of compounds needed to carry out an experiment, but CIBO is general and amenable to any user-defined cost, including logistical availability, synthesizability, number of synthetic steps, structural complexity, safety, time, environmental impact, and sustainability. The framework is also compatible with evolving costs and could, for example, be coupled with an online catalog. Finally, CIBO may also be adapted to planning and optimizing successive reactions and accounting for resource management.^35,48,67^

Overall CIBO is more broadly applicable than standard BO in conditions for which the cost of each experiment extensively varies (*e.g.*, large ligand price differences). We envision the method to be useful for the reaction optimization and development in both traditional and self-driving laboratories.^58,59^

## Data availability

CIBO, an accompanying tutorial, and all the data used in this work are available at https://github.com/lcmd-epfl/cibo.

## Conflicts of interest

There are no conflicts of interest to declare.

## Supplementary Material

DD-003-D4DD00225C-s001
