# Supplementary material for: Cost-informed Bayesian reaction optimization
Source: Digit Discov. 2024 Oct 1;3(11):2289–97. doi: 10.1039/d4dd00225c (PMC11465108; doi:10.1039/d4dd00225c)
Supplement: DD-003-D4DD00225C-s001 [file DD-003-D4DD00225C-s001.pdf]

Supplementary Information (SI) for Digital Discovery.
This journal is © The Royal Society of Chemistry 2024

Supporting Information

Cost-Informed Bayesian Reaction Optimization

Alexandre A. Schoepfer,[†,‡,¶,§] Jan Weinreich,[†,¶,§] Ruben Laplaza,[†,¶] Jerome Waser,[*,‡,¶] and Clemence Corminboeuf[*,†,¶]

†*Laboratory for Computational Molecular Design, Institute of Chemical Sciences and Engineering, École Polytechnique Fédérale de Lausanne (EPFL), 1015 Lausanne, Switzerland*

‡*Laboratory of Catalysis and Organic Synthesis, Institute of Chemical Sciences and Engineering, École Polytechnique Fédérale de Lausanne (EPFL), 1015 Lausanne, Switzerland*

¶*National Center for Competence in Research-Catalysis (NCCR-Catalysis), École Polytechnique Fédérale de Lausanne, 1015 Lausanne, Switzerland*

§*These authors contributed equally to this work*

E-mail: jerome.waser@epfl.ch; clemence.corminboeuf@epfl.ch

Table S1: Glossary of abbreviations

**An**	Aniline
**Be**	Benzamide
**BO**	Bayesian optimization
**BTTP**	2-Methyl-N-(tri(pyrrolidin-1-yl)phosphoranylidene)propan-2-amine
**CC**	Cross-coupling
**CIBO**	Cost-informed Bayesian optimization
**DA**	Direct Arylation
**DBU**	1,8-Diazabicyclo[5.4.0]undec-7-ene
**ECPF2**	Extended connectivity fingerprint
**GPR**	Gaussian process regression
**HTE**	High-throughput experimentation
**ML**	Machine learning
**Mo**	Morpholine
**Pd Catalyst**	Palladium catalyst
**Ph**	Phenethylamine
**qNEI**	Batch noisy expected improvement
**tBuBrettPhos**	Di-tert-butyl(2',4',6'-triisopropyl-3,6-dimethoxy-biphenyl)phosphine
**tBuXPhos**	2-Di-tert-butylphosphino-biphenyl

S1 Data, surrogate models and acquisition functions

The data for the directed arylation (**DA**) and the cross-coupling reactions (**CC**) were taken from the EDBO Github and supplementary information respectively. The **DA** dataset describes the full grid over 12 ligands, 4 bases, 4 solvents, 3 concentrations, and 3 temperatures for a total of 1728 experiments. The **CC** dataset is less exhaustive and does not always use the same resources (*i.e.*, ligands) for the four nucleophiles.

Our implementation of CIBO is based on `BoTorch`.[S1] We use Gaussian process regression (GPR) as our model architecture and batch noisy expected improvement (qNEI) as our default acquisition function.[S1]

$$\mathrm{qNEI}\left(\mathbf{x};\mathcal{D}\right) = \mathbb{E}\left[\left(\max g(\xi) - \max g(\xi_{\mathrm{obs}})\right)_{+} |\mathcal{D}\right]. \tag{S1}$$

In this equation, $\mathbb{E}$ denotes the expectation over the distribution of possible objective function values $g(\xi)$ conditioned on the current data $\mathcal{D}$. The term $\max g(\xi_{\text{obs}})$ represents the maximum observed function value in the dataset, while $\max g(\xi)$ represents the maximum function value in a batch of candidate points ξ. The positive part function $(x)_+ = \max(x, 0)$ ensures that only positive improvements are considered.

GPR is a commonly used surrogate model in Bayesian optimization, which predicts both the output of the objective function and its uncertainty. It is especially powerful owing to its non-parametric nature and ability to model complex, non-linear relationships with a measure of uncertainty. Here, the surrogate models use a Jaccard-Tanimoto kernel as defined in GAUCHE.[S2] The accuracy of the initial GPR models is shown in Figure S1 and S2, noting that their quality improves as the optimization advances by retraining with more data at each iteration.

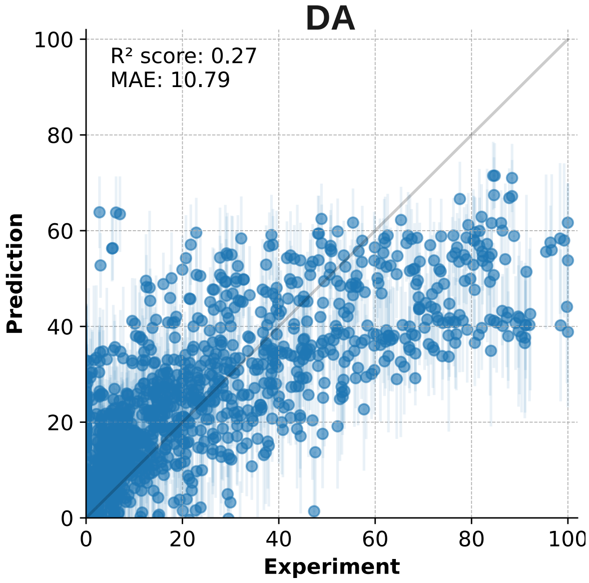

Figure S1: Scatter plots showing yield predictions versus experimental values with $N = 150$ random training points using Gaussian process regression (GPR) with the Jaccard-Tanimoto kernel.[S2] All predictions on the **DA** dataset[S3] are out-of-sample.

Batch noisy expected improvement (qNEI) computes the average expected improvement[S4,S5] over previously observed points by using samples from the joint posterior over the suggested experiments and those previously observed. Note that our approach is not

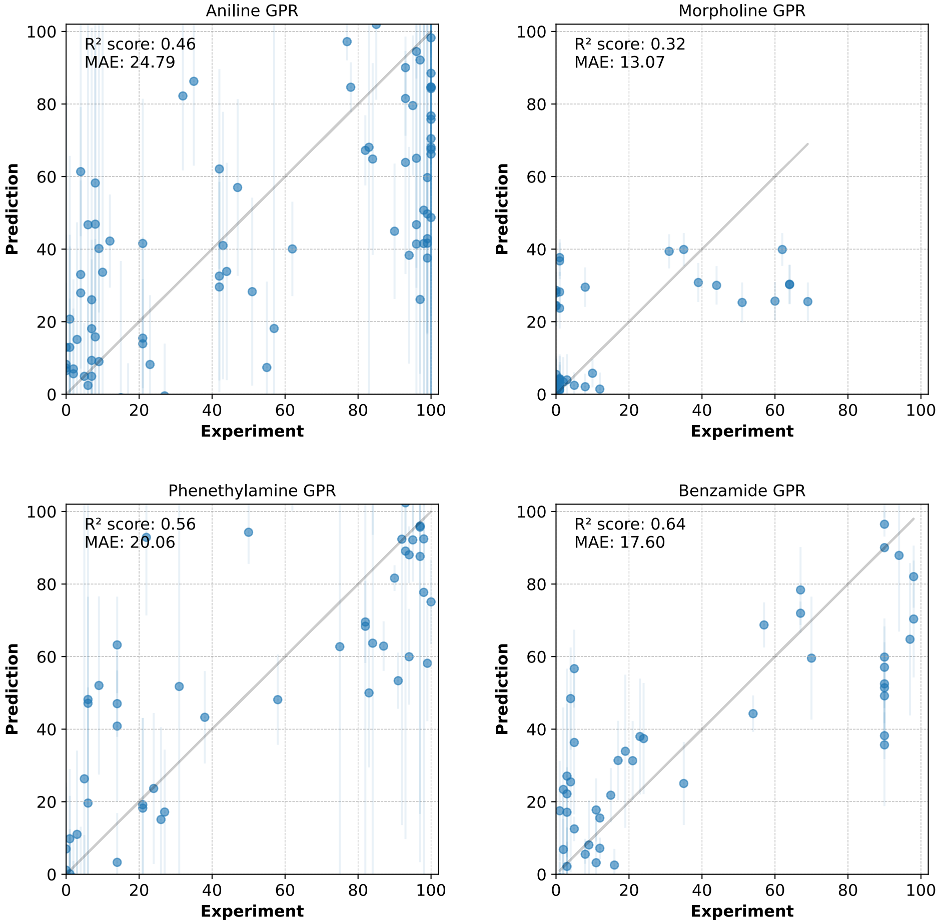

Figure S2: Scatter plots of Gaussian process regression predictions for the **CC** dataset with a fixed training set size of 30 random reaction conditions for fixed nucleophiles (Aniline, Morpholine, Phenethylamine, Benzamide). Predictions (with error bars indicating the uncertainty) are shown on the y-axis, and reference values are shown on the x-axis. The R^2 value and mean absolute error (MAE) are shown on each panel.

restricted to qNEI. Similar expressions could be applied for different acquisition function types, *e.g.*, GIBBON.[S6] Furthermore, the choice of the scaling function S may generally depend on the type of acquisition function. An additional scaling factor could be introduced to guide the optimization toward more or less cost efficiency.

Acquisition functions that account for the exploration–exploitation trade-off can only take non-negative utility values. In other words, adding new data "never hurts", $\alpha \geq 0$.[S7] The modified acquisition function $\tilde{\alpha}$ with costs may take negative values providing a canonical stopping criterion if all remaining experiments have negative utility $\tilde{\alpha} < 0$. Otherwise, the

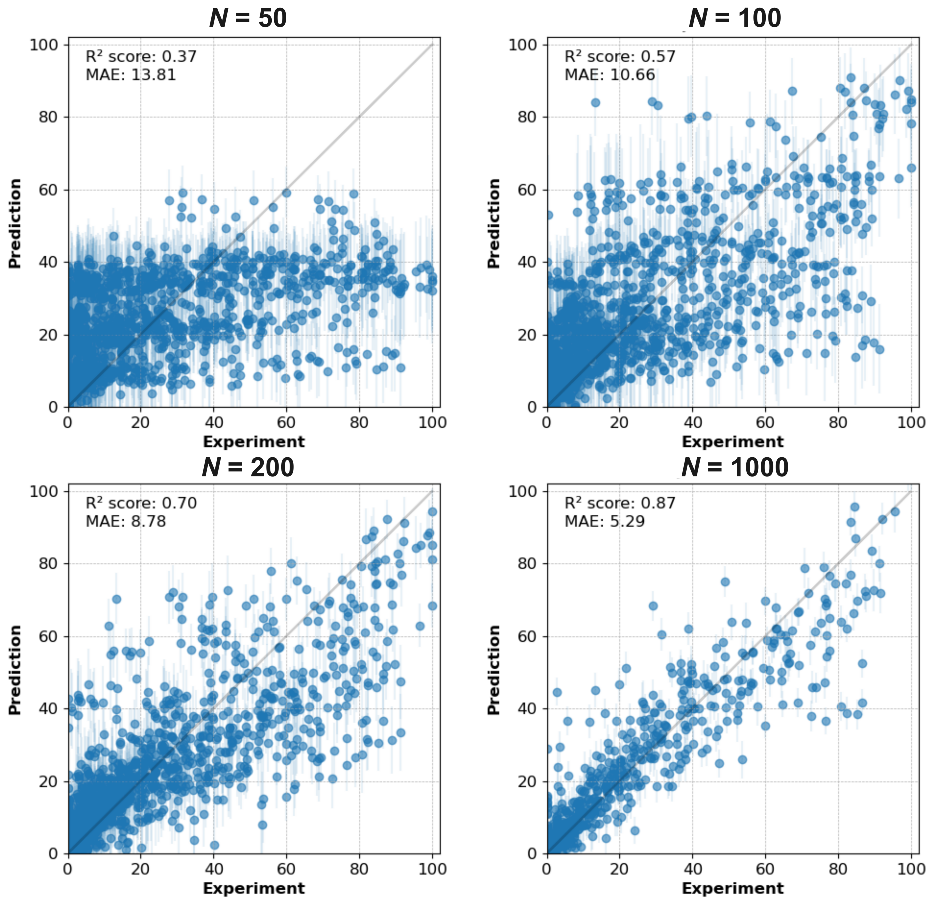

Figure S3: Gaussian process regression predictions with varying training set sizes for the Direct Arylation reaction dataset. Scatter plots show yield predictions versus experimental values for different training set sizes ($N = 50, 100, 200, 1000$) random training points using Gaussian process regression (GPR) with the Jaccard-Tanimoto kernel. Each plot includes the R^2 score and mean absolute error (MAE) to assess the model performance.

optimization can be halted when a target yield has been obtained.

In this next experiment, we look at how the model improves yield prediction by increasing the amount of training data. As shown in the scatter plots for the **DA** dataset (Figure S3) the model improves with larger training set sizes, starting with an $R^2 = 0.37$ for $N = 50$ training points and reaching $R^2 = 0.87$ for $N = 1000$. For a more systematic analysis, the mean absolute error (MAE) for yield prediction is given as a function of training set size (see Figure S4) on a double logarithmic scale. The results confirm that the error decreases systematically with increasing N.

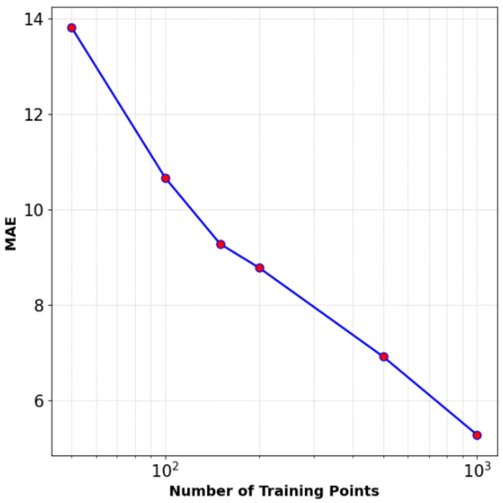

Figure S4: Learning curve, mean absolute error of the yield prediction from the Gaussian process regression model as a function of training set size, using a non-intersecting set of random points from the direct arylation dataset as training and test set.

S2 Reaction representations

To represent a chemical compound $\mathbf{x}$, we use extended connectivity fingerprints[S8] of radius two (ECFP2) with 512 features per molecule. The input $\mathbf{X}$ for our models includes these ECFP2 descriptors along with reaction conditions such as temperature T and concentration c when available, forming a concatenated representation vector. For instance, a single experiment of the **DA** dataset is represented as

$$\mathbf{X} = [\mathbf{x}_{\text{ligand}}, \mathbf{x}_{\text{solvent}}, \mathbf{x}_{\text{base}}, T, c], \tag{S2}$$

and an experiment of the **CC** dataset – as

$$\mathbf{X} = [\mathbf{x}_{\text{ligand}}, \mathbf{x}_{\text{solvent}}, \mathbf{x}_{\text{base}}, c_{\text{base}}, eq._{\text{base}}, T, t], \tag{S3}$$

where $eq.$ are equivalents, and t is the time.

S3 Price sources

The cost of chemicals was previously reported for the **DA** dataset.[S9]Prices, for the **CC** dataset, were taken from various Swiss providers (look-up date: Jan, 9th 2024). Exact sources and prices are listed on the code repository. Samples closest or equal to 1 g were used as a reference and were converted to dollars per gram ($/g) for consistency. Realistically, the full sample price should be taken. Moreover, depending on the molecular weight and stoichiometry of chemicals, dollars per mole may be a more appropriate measure in some cases. For simplicity, we also convert CHF to USD with a conversion rate of 1. The process of obtaining prices could be automatized by using recently developed tools.[S10,S11]

S4 Weight factor for including costs

In Figure S5, we compare the effect of choosing different weighting factors λ to include the cost consideration into the yield optimization of CIBO for the **DA** dataset. The initialization and surrogate model are the same type as in the main text. A large λ means putting more weight on lowering the cost of acquiring new compounds, a smaller value means the opposite.

In general, the choice of λ depends on user preferences. We find that simply setting $\lambda = 1$ worked well for the five retrospective studies considered here. For prospective studies, we recommend comparing the suggestions given by both BO and CIBO and eventually adapting λ.

As expected, using $\lambda = 2.0$, twice the default value, for the cost weights yields much smaller cumulative costs. However, while the costs are less than a fourth of a random search, in this case, the best yield found is not much better than random. $\lambda = 1.0$ is equivalent to the result shown in the manuscript.

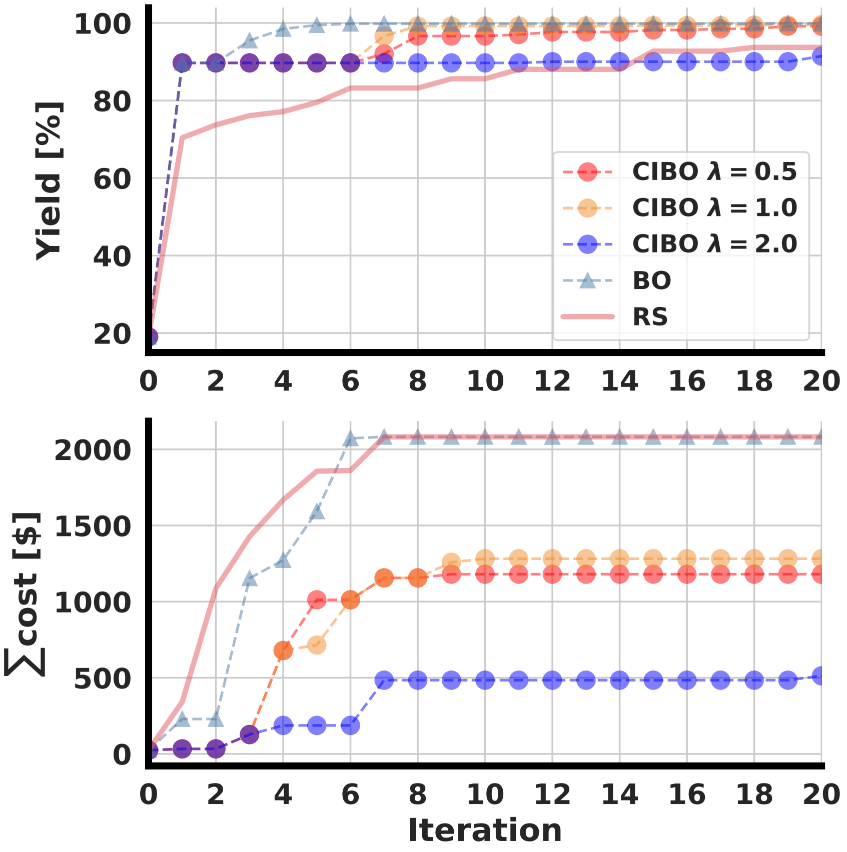

Figure S5: Comparison of cost-informed Bayesian optimization with weighting factor (Equation 4 in paper) $\lambda = 1.0$ (CIBO, orange). CIBO results for $\lambda = 0.5$ and $\lambda = 2.0$ are shown with red and blue circles, respectively. Standard BO (blue triangles) for the **DA** dataset and Random Search (RS) (red line) are also shown. Average curves over five runs are presented. The best-obtained yield in each batch iteration is shown in the top panel, and the total cost to acquire the ligands is shown in the bottom panel.

S5 Other initializations

To further investigate and compare the performance of CIBO and BO, several additional initialization experiments were conducted. The results of these experiments, using different initialization strategies, are shown in this section.

In Figure S6 we analyzed how starting from different ligands alters the optimization for the **DA** dataset. For each of the 100 optimization runs, one of the 12 ligands was randomly chosen and the 144 experiments using that ligand were used to initialize the surrogate model. Overall, no significant changes are observed compared to Figure 3 (main text) apart from

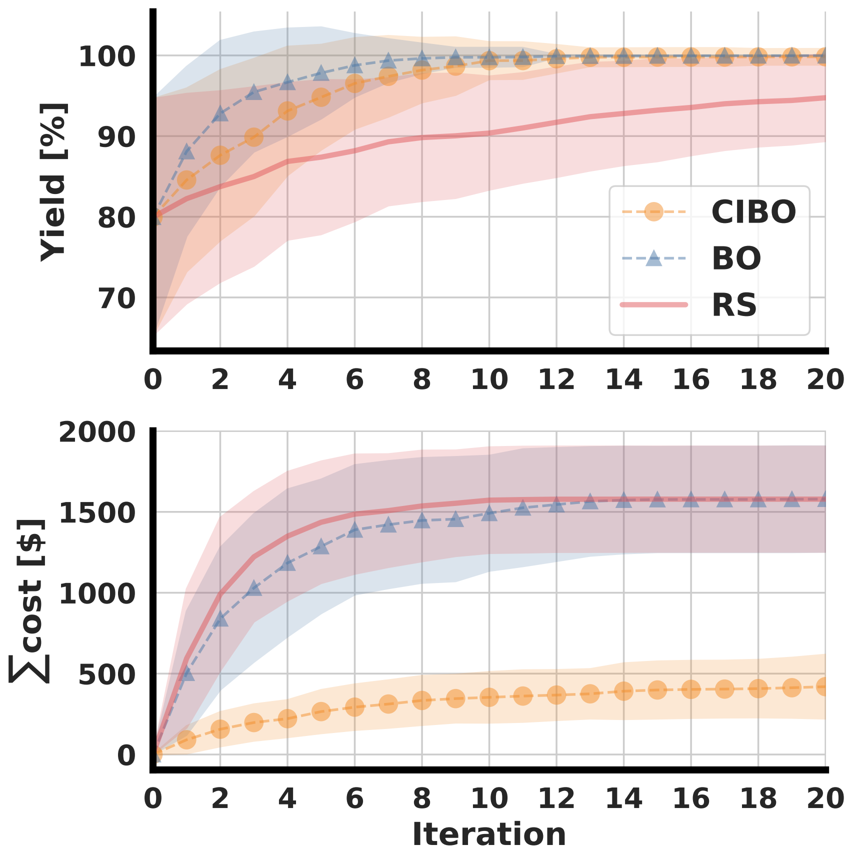

Figure S6: Comparison of CIBO (orange), standard BO (blue), and random sampling (RS, red) for the **DA** dataset, averaged over 100 runs and batch size of $q = 5$. Each optimization was initialized with one of the 12 ligands chosen randomly and its 144 corresponding experiments.

random sampling performing worse. Given that each initialization starts with a different ligand, the cost curve in Figure S6 is initiated at $0 and only the cost of the following newly acquired compound is then added to the total cost.

As shown in Figure 3, random sampling consistently underperforms compared to BO, yet high yield values are obtained due to the multiple combinations leading to high yields in the **DA** dataset. In Figure S6 where different ligands are chosen initially, random sampling performs even worse on average. The performance starts around 80% yield, which corresponds to the average of all data points for **DA**.

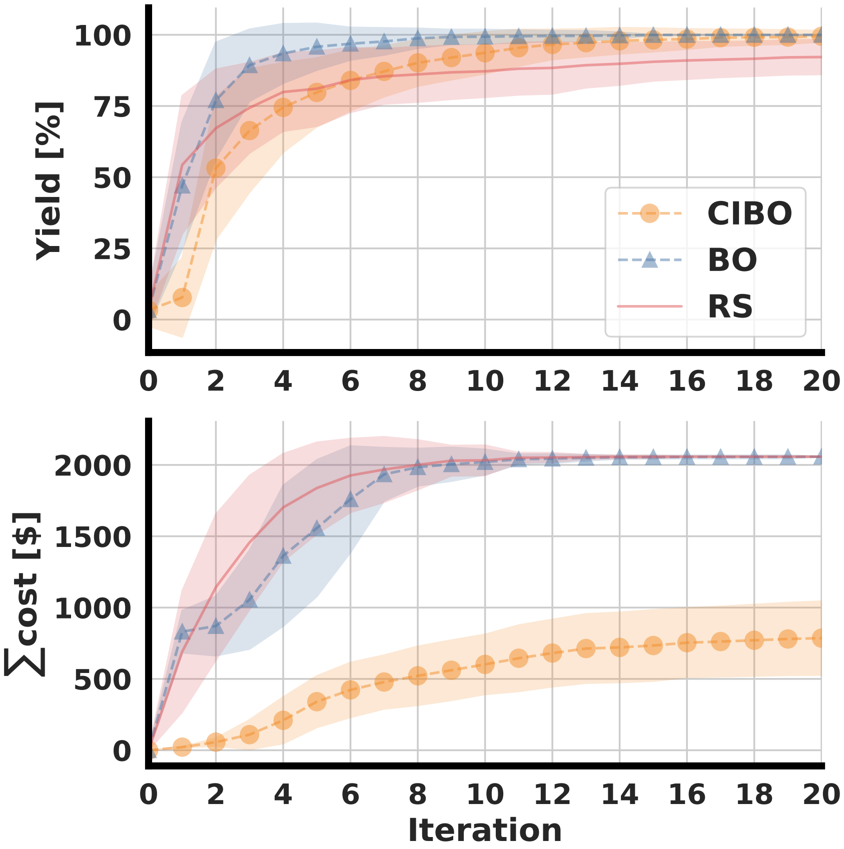

Figure S7: Comparison of CIBO (orange), standard BO (blue), and random sampling (RS, red) for the **DA** dataset, averaged over 100 runs. Each optimization was initialized using 10 random experiments with the worst ligand (*i.e.*, the ligand which given all other reaction conditions has the lowest yield) and a batch size of $q = 5$.

Figure S7 shows runs initialized with the worst ligand from **DA**. In comparison to Figure 3 (main text), only 10 experiments are chosen randomly instead of the previous 144. This reflects a more realistic scenario, where less experiments are available at the onset of optimization. Both BO and CIBO take more experiments to reach yields of 99% but CIBO manages to spend even less than when starting with 144 experiments.

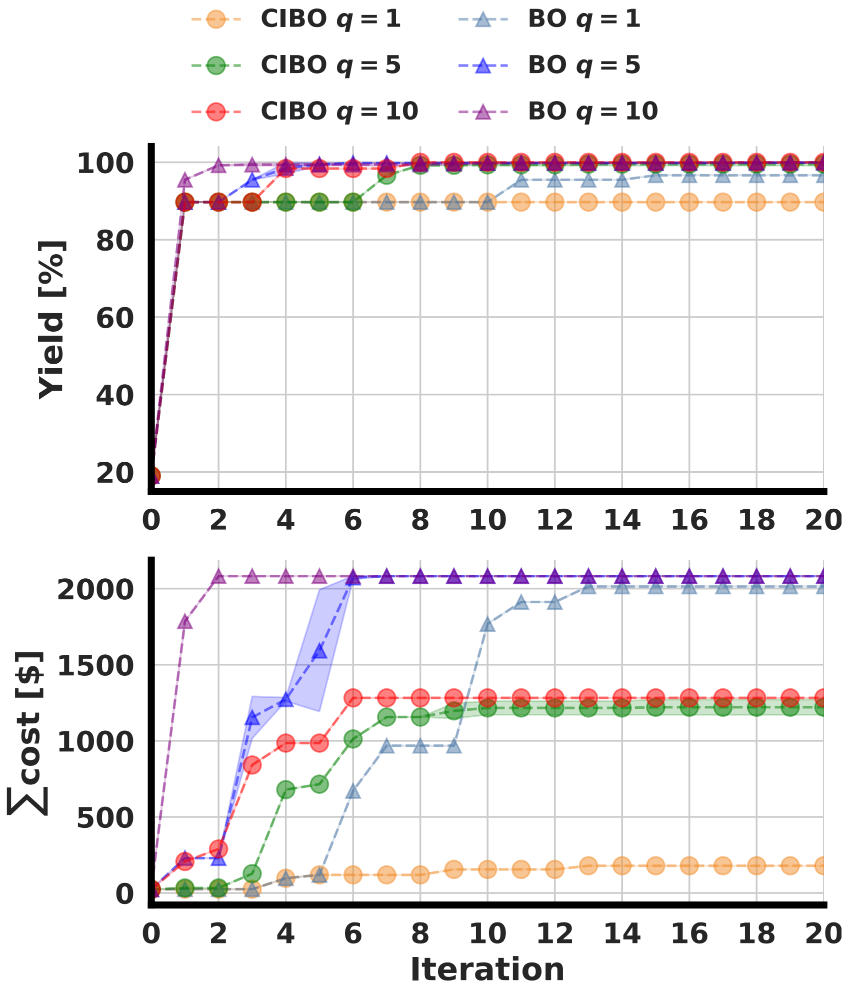

Figure S8: Comparison of CIBO versus BO for different batch sizes $q = 1, 5, 10$ using the worst ligand initialization as in the main text. The maximal yield as well as the budget spent for the discovery campaign are shown in the top and bottom panel respectively.

We also explore the effect of different batch sizes q (shown Figure S8) Since more data is added at each iteration to train the reference model, increasing the batch sizes from 5 to 10 results in faster convergence of both BO and CIBO. CIBO appears to become more "greedy" if the batch size is decreased and tends to fully explore all "cost-free" dimensions. This behavior however can be adjusted by changing the weighting factor λ (see SI section S4).

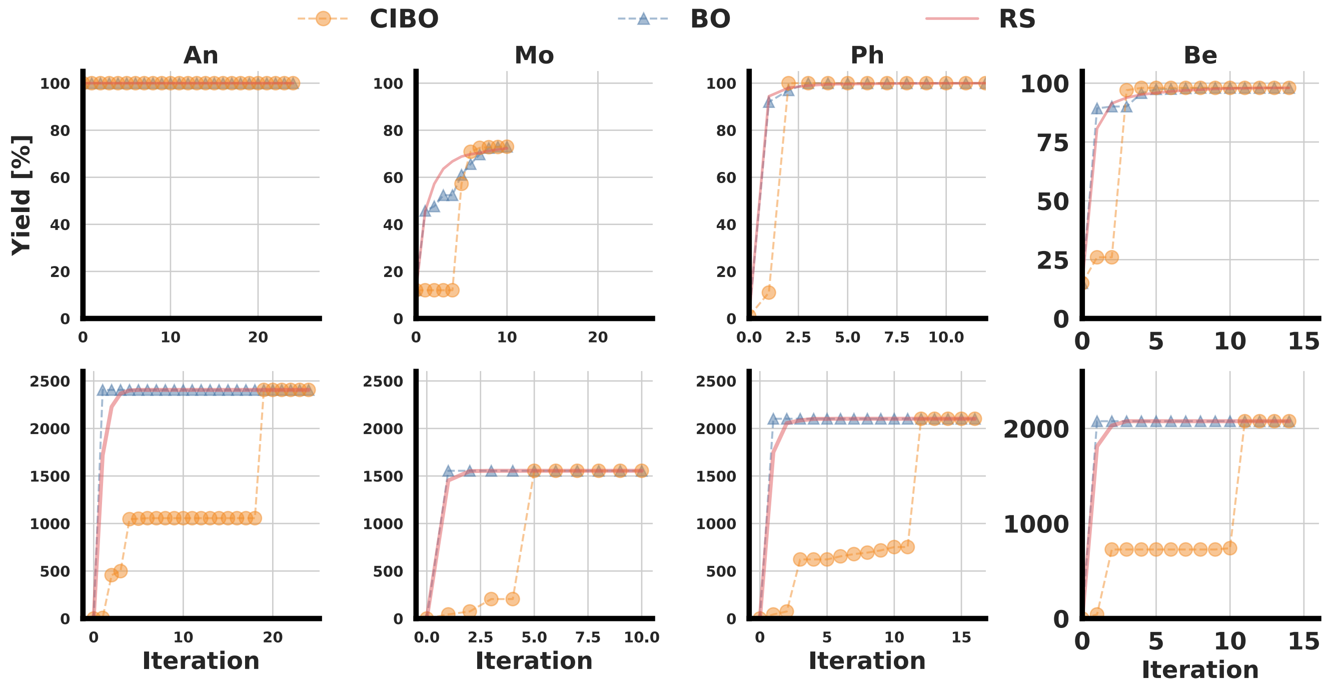

Figure S9: Yield optimization for the **CC** dataset with four different nucleophiles (**An**, **Mo**, **Ph**, **Be**) starting with the cheapest available ligand. We compare cost-informed Bayesian optimization (CIBO, orange), Bayesian optimization (BO, blue), and random sampling (RS, red). Average curves over 100 runs are shown. Error bars are shown but not visible because the optimization is completely determined by the initialization. The top row shows the best yield found as a function of the batch iteration, and the bottom row displays the cumulative costs.

Figure S9 and S10 show both initializations used for Figure 5 (main text). Starting with the cheapest ligand for Aniline leads to a perfect yield even prior to optimization.

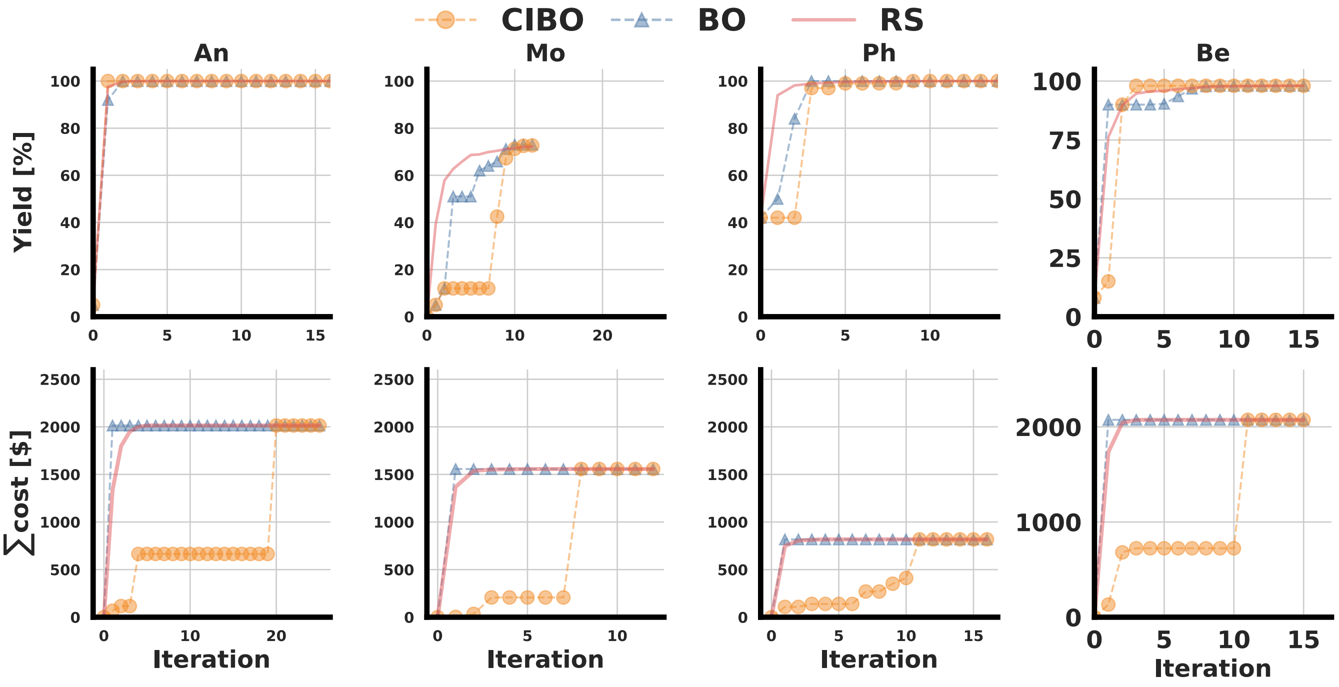

Figure S10: Yield optimization for the **CC** dataset with four different nucleophiles (**An**, **Mo**, **Ph**, **Be**). Runs are initialized with the worst ligand. We compare cost-informed Bayesian optimization (CIBO, orange), Bayesian optimization (BO, blue), and random sampling (RS, red). Average curves over 100 runs are shown. Error bars are shown but not visible because the optimization is completely determined by the initialization. The top row shows the best yield found as a function of the batch iteration, and the bottom row displays the cumulative costs.

References

[S1] Balandat, M.; Karrer, B.; Jiang, D. R.; Daulton, S.; Letham, B.; Wilson, A. G.; Bakshy, E. BoTorch: A framework for efficient Monte-Carlo Bayesian optimization. Adv. Neural Inf. Process. Syst. 2020; pp 21524–21538.

[S2] Griffiths, R.-R. et al. GAUCHE: A library for Gaussian processes in chemistry. *arXiv preprint* **2023**, arXiv:2212.04450.

[S3] Shields, B. J.; Stevens, J.; Li, J.; Parasram, M.; Damani, F.; Alvarado, J. I. M.; Janey, J. M.; Adams, R. P.; Doyle, A. G. Bayesian reaction optimization as a tool for chemical synthesis. *Nature* **2021**, *590*, 89–96.

[S4] Močkus, J. On Bayesian methods for seeking the extremum. Optimization Techniques IFIP Technical Conference. 1975; pp 400–404.

[S5] Jones, D. R.; Schonlau, M.; Welch, W. J. Efficient global optimization of expensive black-box functions. *J. Glob. Optim.* **1998**, *13*, 455–492.

[S6] Moss, H. B.; Leslie, D. S.; Gonzalez, J.; Rayson, P. GIBBON: General-purpose Information-Based Bayesian OptimisatioN. *J. Mach. Learn. Res.* **2021**, *22*, 1–49, Submitted 2/21; Revised 9/21; Published 9/21.

[S7] Garnett, R. *Bayesian Optimization*; Cambridge University Press, 2023.

[S8] Rogers, D.; Hahn, M. Extended-connectivity fingerprints. *J. Chem. Inf. Model.* **2010**, *50*, 742–754.

[S9] Torres, J. A. G.; Lau, S. H.; Anchuri, P.; Stevens, J. M.; Tabora, J. E.; Li, J.; Borovika, A.; Adams, R. P.; Doyle, A. G. A multi-objective active learning platform and web app for reaction optimization. *J. Am. Chem. Soc.* **2022**, *144*, 19999–20007.

[S10] Sorkun, M. C.; Saliou, B.; Er, S. ChemPrice, a Python package for automated chemical price search. *ChemRxiv preprint* **2024**, chemrxiv–2024–1bxgg.

[S11] Sanchez-Garcia, R.; Havasi, D.; Takács, G.; Robinson, M. C.; Lee, A.; von Delft, F.; Deane, C. M. CoPriNet: Graph neural networks provide accurate and rapid compound price prediction for molecule prioritisation. *Digital Discovery* **2023**, *2*, 103–111.
